# *Exocarpium Citri Grandis*‐Derived Extracellular Vesicle‐Like Particles for Accelerating Wound Healing via Regulating the Protein Expression on the VEGF/AKT Signaling Pathway

**DOI:** 10.1002/fsn3.71472

**Published:** 2026-01-28

**Authors:** Yingjie Xiong, Zanxiang Luo, Jingxiu Zhao, Chengshi Fu, Yujia Song, Jinyong He, Jiahui Gao, Zejie Su, Lie Liu, Xiangyun Teng, Jianhua Xu

**Affiliations:** ^1^ Department of Laboratory Medicine Shunde Hospital of Guangzhou University of Chinese Medicine Foshan China; ^2^ Department of Clinical Laboratory Maoming Hospital of Guangzhou University of Chinese Medicine Maoming China; ^3^ Translational Research Center for Traditional Chinese Medicine Maoming Hospital of Guangzhou University of Chinese Medicine Maoming China

**Keywords:** *Exocarpium Citri Grandis*, plant‐derived extracellular vesicle‐like particles, protein kinase B (AKT), vascular endothelial growth factor A (VEGFA), wound healing

## Abstract

*Exocarpium Citri Grandis* (ECG) is a plant endemic to Huazhou City, Guangdong Province, China. It is utilized both as a health food and in traditional medicine. Recently, *Exocarpium Citri Grandis*‐derived extracellular vesicle‐like particles (ECG‐EVLP) have been isolated from ECG. Given the significant advantages of plant‐derived extracellular vesicle‐like particles (P‐EVLP), these particles from various sources have been investigated for their potential in wound healing applications, reducing wound area in vitro or in vivo models. Although the anti‐inflammatory and antioxidant activities of ECG‐EVLP have been established in previous studies, their role in skin wound healing remains unexplored. Our findings indicate that ECG‐EVLP can effectively promote wound healing. In vivo, wound healing was significantly improved in the ECG‐EVLP group compared with the PBS group on Days 3, 7, 11, and 14. In vitro, ECG‐EVLP significantly enhanced L929 cell proliferation at all concentrations (150, 300, and 450 μg/mL) after both 24 and 48 h. For HaCat cells, proliferation increased at the two high concentrations (300 and 450 μg/mL) after 24 h and extended to all concentrations, including all concentrations (150, 300, and 450 μg/mL), after 48 h. The activation of the VEGF/AKT signaling pathway, together with the inhibition of the mitochondrial apoptosis pathway, is likely the underlying mechanism. This interplay promotes cell proliferation, migration, and collagen production, thereby accelerating wound healing following ECG‐EVLP stimulation. Additionally, sphingosine and naringin might be the effective components of ECG‐EVLP in promoting wound healing.

## Graphical abstract

Our findings indicate that ECG‐EVLP can effectively promote wound healing and the activation of the VEGF/AKT signaling pathway in vitro and in vivo, coupled with the inhibition of the mitochondrial apoptosis pathway in cells following ECG‐EVLP stimulation might be its underlying mechanism. Additionally, sphingosine and naringin of ECG‐EVLP are identified as potential active components that might contribute to promoting wound healing.

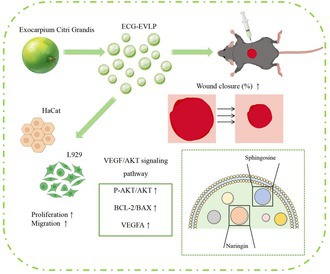

**Abbreviations**
AKT: protein kinase B
ANOVA: analysis of variance
ATCC: American Type Culture Collection
BAX: BCL‐2‐associated X protein
BCA: bicinchoninic acid assay
BCL‐2: B‐cell lymphoma‐2
BRCA1: breast cancer 1
DCFH‐DA: 2′,7′‐dichlorodihydrofluorescein diacetate
DMEM: Dulbecco's modified Eagle medium
ECG: *Exocarpium Citri Grandis*
ECG‐EVLP: *Exocarpium Citri Grandis*‐derived extracellular vesicle‐like particles
ECM: extracellular matrix
ELISA: enzyme‐linked immunosorbent assay
eNOS: endothelial nitric oxide synthase
FBS: fetal bovine serum
FITC: fluorescein isothiocyanate
FNVs@RAPA: fusion extracellular nano‐vesicular system carrying rapamycin
GO: gene ontology
HaCat: human keratinocytes
HE: hematoxylin–eosin
IL‐1β: interleukin‐1β
IL‐6: interleukin‐6
IRI: ischemia–reperfusion injury
KEGG: Kyoto Encyclopedia of Genes and Genomes
L929: NCTC clone 929
MEVs: milk‐derived extracellular vesicles
NTA: nano tracking analysis
P‐AKT: phospho‐protein kinase B
PBS: phosphate buffer saline
P‐EVLP: plant‐derived extracellular vesicle‐like particles
PVDF: polyvinylidene fluoride
ROS: reactive oxygen species
SDS‐PAGE: sodium dodecyl sulfate‐polyacrylamide gel electrophoresis
STR: short tandem repeat
TBST: tris‐buffered saline with Tween 20
TEM: transmission electron microscopy
TNF‐α: tumor necrosis factor‐α
VEGFA: vascular endothelial growth factor A
VEGFR2: vascular endothelial growth factor receptor 2

## 1 Introduction

As the body's largest organ, the skin performs multiple functions, including temperature regulation, sensation (such as touch, pain, and pressure), and serves as a protective barrier against mechanical stress and pathogens. Skin damage can result from various conditions, including surgery, trauma, burns, and complications of diabetes. Wound healing proceeds through four distinct but overlapping stages: hemostasis, inflammation, proliferation, and remodeling. After damage, keratinocytes migrate from the basal layer to the wound site and proliferate to repair the epidermal layer (Rodrigues et al. 2019). Concurrently, fibroblasts migrate to the wound area, producing cytokines, growth factors, and extracellular matrix (ECM) essential for rebuilding the dermis (Wilkinson and Hardman 2020). During these stages, multiple signaling pathways such as MAPK, PI3K/AKT, TGF‐β, Wnt, NF‐κB, Notch, and Hippo signaling pathway (Liu and Fang 2025) are involved in the proliferation and repair of skin cells (Wilkinson and Hardman 2020).

In recent years, numerous studies have demonstrated that the small size and negative charge of extracellular vesicles, along with their excellent physicochemical stability across various pH and temperature conditions, facilitate their efficient penetration into target cells (Li et al. 2024). Previous researches have shown that some animal‐derived exosomes promote wound healing to varying degrees by modulating immune cells, endothelial cells, and skin fibroblasts at different stages of tissue repair (Shao et al. 2024; Yu et al. 2024; Zhang, Huang, et al. 2024). However, the widespread use of animal‐derived exosomes is limited due to their inherent immunogenicity, challenges in sourcing, susceptibility to degradation, and high storage costs (Cheng and Hill 2022; Feng et al. 2023). Compared to animal‐derived exosomes, P‐EVLP offer significant advantages. They are readily available in large quantities, exhibit low immunogenicity, are cost‐effective to produce, and demonstrate remarkable stability (Feng et al. 2024; Wu et al. 2024). In terms of function, P‐EVLP have no less therapeutic potential than animal‐derived exosomes in wound healing, tumors, nervous system diseases, digestive system diseases, colitis, pulmonary diseases, viral‐transmitted diseases, and oral diseases (Rehman et al. 2025). P‐EVLP derived from sources such as wheat (Şahin et al. 2019), grapefruit (Savcı et al. 2021), *Aloe vera* (Kim and Park 2022; Ramírez et al. 2024), tomato (Daniello et al. 2024), *Morus* sp. (Mulberry) (Garrett et al. 2024), *Physalis peruviana* (Natania et al. 2025), *Baeckea frutescens* L. (Safwan Kamarazaman et al. 2024), *Opuntia ficus‐indica* (Valentino et al. 2024), and Dendrobium (Tu et al. 2024) have been investigated for their potential in wound healing applications, showing promising results.

*Exocarpium Citri Grandis* (ECG) is a traditional Chinese medicinal herb with a rich history of use, originating from Huazhou City in Guangdong Province. For thousands of years, its exocarp and whole fruit have been clinically employed as effective antitussive and expectorant agents. Additionally, its peel and flowers are traditionally used to prepare healthful teas and beverages. In 2024, ECG was officially approved as a medicinal and food homologous substance in China, in accordance with the Food Safety Law of the People's Republic of China and its implementing regulations. This marked a significant milestone, formally classifying ECG under the food category while acknowledging its long‐standing medicinal use. Over the centuries, ECG has been widely used to treat conditions such as coughs and phlegm. Modern scientific researches have further validated its therapeutic potential, demonstrating anti‐inflammatory, antimicrobial, antioxidant, and anti‐atherosclerotic properties. Recent studies have shown that naringin, the main component of ECG, can improve wound healing by upregulating MMP and VEGF protein expression (Yen et al. 2022).

In a study of Sun Yat‐sen University, ECG‐EVLP was used to create a novel, ROS‐reactive, multifunctional fusion extracellular nano‐vesicular system carrying rapamycin (FNVs@RAPA) (Lu et al. 2024). This innovative formulation was developed to tackle early ischemia–reperfusion injury (IRI) and Ly6C + Ly6G‐inflammatory macrophage‐mediated rejection in heart transplantation. Previous studies have shown that anti‐inflammatory and antioxidant activities can promote wound healing, so it is reasonable to believe that ECG‐EVLP can promote wound healing by reducing tissue damage and scavenging free radicals; it creates a favorable microenvironment for cell repair and regeneration (Mohite et al. 2025). While the anti‐inflammatory and antioxidant activities of ECG‐EVLP have been established in the study of Sun Yat‐sen University, the specific role and mechanism of ECG‐EVLP in skin wound healing has yet to be investigated.

**FIGURE 1:**
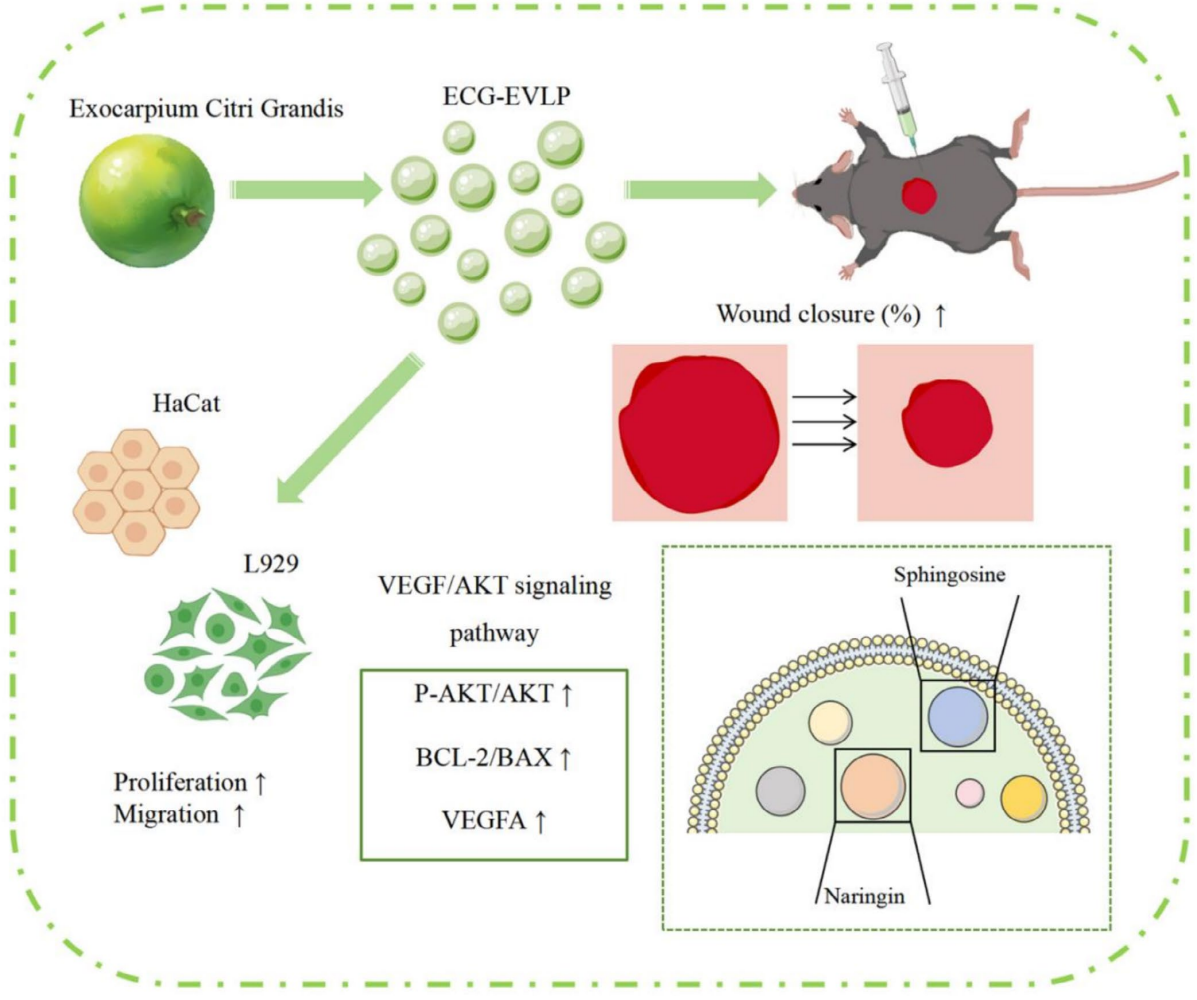
Graphical summary of ECG‐EVLP accelerating wound healing. A graphical summary of our findings indicates that ECG‐EVLP promotes wound healing by activating the VEGF/AKT signaling pathway and suppressing the mitochondrial apoptosis pathway in both in vitro and in vivo models.

From previous studies, we learned about the potential of P‐EVLP in promoting wound healing, the roles of VEGF and AKT signaling pathways in wound healing, and the anti‐inflammatory and anti‐oxidative activities of ECG‐EVLP. Therefore, our study aimed to elucidate the role of ECG‐EVLP in wound healing and potential mechanisms by upregulating the VEGF/AKT signaling pathway and inhibiting the mitochondrial apoptosis pathway through in vivo and in vitro experiments (Figure 1).

## 2 Experimental Materials and Methods

### 2.1 Isolation and Purification of ECG‐EVLP

The fresh ECG fruits utilized in this study were sourced from Huazhou, Maoming City, located in Guangdong Province. No special institutional approvals or regulatory permissions were necessary for conducting research with these botanical specimens. The extraction and refinement protocol for ECG‐EVLP was carried out through a sequential procedure comprising multiple critical stages.

#### 2.1.1 Cell Disruption Procedure

Fresh citrus fruits nearing full maturity (4–6 cm diameter) underwent thorough cleaning before being sliced into uniform fragments. The prepared samples were subsequently processed using a mechanical homogenizer to extract the liquid content. The resulting citrus extract was then subjected to filtration through a 440‐mesh sieve to remove coarse particulates, followed by aliquot distribution into 50 mL conical centrifugation vessels.

#### 2.1.2 Gradient Centrifugation Pretreatment

The clarified juice underwent sequential centrifugation steps under refrigerated conditions (4°C) using progressively increasing forces—initial separation at 1000 *g* (10 min), followed by 3000 *g* (20 min), then 4500 *g* (20 min), subsequently 6500 *g* (30 min), and final clarification at 10,000 *g* (60 min). After each centrifugation cycle, the liquid supernatant was carefully collected. The final supernatant was filtered through 0.45 μm membrane filters to remove particulate impurities.

#### 2.1.3 Polyethylene Glycol‐Based Precipitation Isolation

For every 20 mL of supernatant, 5 mL of prepared precipitant solution (10% PEG concentration) was added and thoroughly mixed. The mixture was subsequently maintained under refrigeration at 4°C for 8 h. Following precipitation, the solution was subjected to centrifugation at 12,000 *g* for 60 min under chilled conditions (4°C) to isolate the nanoparticle precipitates. The PEG concentration and incubation duration were optimized based on previous studies (Zhao et al. 2022).

#### 2.1.4 Filtration and Purification

**FIGURE 2:**
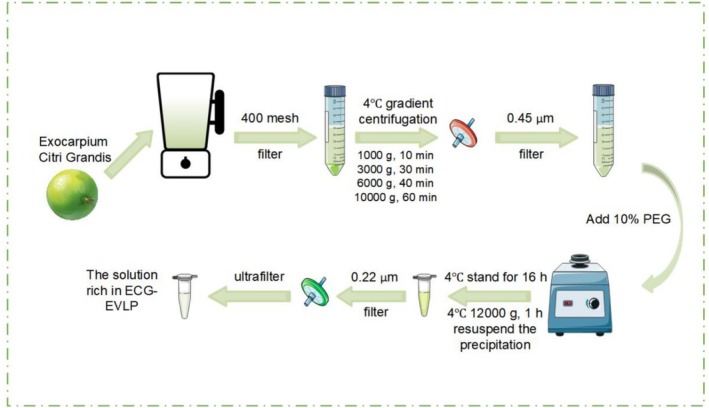
Schematic diagram of the isolation and purification process of ECG‐EVLP. An improved detailed isolation and purification process of ECG‐EVLP.

The nanoparticle suspension in phosphate‐buffered saline (PBS) underwent sequential processing through a sterile 0.22 μm membrane filtration system for purification and sterilization purposes. This purification protocol yielded ECG‐EVLP through collection of the clarified filtrate (Figure 2). The ECG‐EVLP solution was stored temporarily in a refrigerator at −80°C after protein quantification using the bicinchoninic acid assay (BCA) kit (Servicebio, China).

### 2.2 The Surface Morphology of ECG‐EVLP

A 10‐μL aliquot of the sample was carefully applied to the copper grid, ensuring the supporting membrane surface made direct contact with the liquid sample. Subsequently, 1‐μL uranyl acetate solution was introduced to the grid (establishing contact between sample and stain), allowed to dry at room temperature. ECG‐EVLP morphology was examined using transmission electron microscopy (TEM) (Hitachi HT7700, Japan) for detailed structural analysis based on three biological replicates. The acceleration voltage was 80 kV, and the amplification range was 40,000 times.

### 2.3 Determination of Particle Size of ECG‐EVLP

To assess the particulate dimensions of ECG‐EVLP, the sample solution underwent appropriate dilution (1:1000 ratio) and was maintained at ambient temperature. The diluted particle concentration is 4.4 × 10^7^–4.8 × 10^7^ particles/mL. Particle characterization was performed through nanoparticle tracking analysis (NTA) using a Particle Metrix Zetaview‐PMX120‐Z instrument (Germany) based on three biological replicates. The instrument quantified particle dimensions by measuring the Brownian motion of suspended particles under laser illumination.

### 2.4 Untargeted Metabolomics Analysis of ECG‐EVLP

#### 2.4.1 Sample Preparation

The lipid extraction procedure begins by transferring ECG‐EVLP samples into a 2 mL centrifuge tube containing 100 mg of glass beads. To each tube, 1000 μL of a pre‐cooled acetonitrile:methanol:water (2:2:1, v/v/v) solution is accurately added, followed by vortex mixing for 30 s. For particle disruption, the tube is placed into a 2 mL adapter and immersed in liquid nitrogen for rapid freezing for 5 min. The frozen sample is then thawed at room temperature. The adapter containing the tube is installed into a tissue grinder and homogenized at 60 Hz for 2 min. This freeze–thaw–homogenization cycle is repeated twice for complete lysis. Following homogenization, the sample is centrifuged at 12,000 rpm for 10 min at 4°C. The entire supernatant is carefully collected and transferred to a new 2 mL centrifuge tube. The supernatant is then concentrated to complete dryness using an appropriate concentrator. For LC–MS analysis, the dried lipid extract is accurately reconstituted in 300 μL of a solution consisting of acetonitrile and 4 ppm 2‐amino‐3‐(2‐chloro‐phenyl)‐propionic acid in 0.1% formic acid (1:9, v/v). The redissolved sample is finally filtered through a 0.22‐μm membrane and transferred to a vial for LC–MS detection. For QC samples, pooled samples were used, that is, parts of the extracted samples to be tested were mixed.

#### 2.4.2 Liquid Chromatography Conditions

Chromatographic separation was conducted on a Vanquish UHPLC platform (Thermo Fisher Scientific, USA) equipped with an ACQUITY UPLC HSS T3 column (2.1 × 100 mm, 1.8 μm particle size; Waters, Milford, MA). The analytical column was maintained at 40°C throughout the analysis. For positive ion mode ESI‐MS detection, the mobile phase comprised two components: (A2) aqueous solution containing 0.1% formic acid (v/v) and (B2) acetonitrile with 0.1% formic acid (v/v). The gradient program was set as: 0–1 min at 8% B2; 1–8 min with a linear increase from 8% to 98% B2; maintained at 98% B2 from 8 to 10 min; followed by a rapid return to 8% B2 between 10 and 10.1 min; and equilibration at 8% B2 from 10.1 to 12 min.

The LC‐ESI (−)‐MS analytical parameters were established as follows: mobile phase B3 consisted of acetonitrile while mobile phase A3 contained 5 mM ammonium formate solution. The gradient elution program operated under these conditions: 0–1 min: 8% B3; 1–8 min: linear gradient from 8% to 98% B3; 8–10 min: isocratic elution at 98% B3; 10–10.1 min: rapid return to initial conditions (8% B3); 10.1–12 min: system equilibration at 8% B3. A sample injection volume of 2 μL was maintained with a constant flow rate of 0.3 mL/min throughout the analysis.

#### 2.4.3 Mass Spectrum Conditions

Metabolite detection via mass spectrometry was performed on a Q Exactive system (Thermo Fisher Scientific, USA) equipped with an electrospray ionization source. The experimental setup involved simultaneous acquisition of MS1 and MS/MS spectra through Full MS‐ddMS2 mode with data‐dependent fragmentation. Instrumental parameters were configured as follows: sheath gas maintained at 40 arbitrary units, auxiliary gas flow rate set to 10 arb, and ionization voltages adjusted to +3.50 kV for positive mode along with −2.50 kV for negative ion detection.

The electrospray ionization (ESI) parameters were optimized with 50 kV applied to both positive and negative ionization modes. The capillary heating system maintained a 325°C operational temperature. Primary mass spectrometry (MS1) scanning operated within the m/z 100–1000 range with 70,000 full width at half maximum resolution. Each acquisition cycle included 10 data‐dependent secondary scans, with MS/MS analysis achieving 17,500 FWHM resolution. Collision‐induced dissociation utilized 30 eV normalized energy, while dynamic exclusion duration followed instrument‐optimized settings.

### 2.5 Untargeted Lipidomics Analysis of ECG‐EVLP

#### 2.5.1 Chromatographic Conditions

Chromatographic analysis was performed on an ACQUITY UPLC BEH C18 column (2.1 × 100 mm, 1.7 μm; Waters) with temperature parameters set at 50°C for the column and 8°C for the autosampler. The gradient elution program consisted of multiple phases: initial 70% A2 decreasing to 57% over 0–5 min, followed by rapid adjustment to 50% A2 at 5.1 min. From 5.1 to 14 min, the mobile phase transitioned linearly from 50% to 30% A2, maintaining this concentration until 14.1 min. Subsequent phases included progressive reduction to 1% A2 by 21 min, sustained until 24 min, followed by rapid re‐equilibration to initial conditions (70% A2 at 24.1 min) maintained through 28 min. The mobile phase composition remained constant at acetonitrile:water (60:40) containing 0.1% formic acid throughout the separation process.

The mobile phase was composed of two components: 0.1% formic acid with 10 mM ammonium formate in water (A2) and a 90:10 mixture of isopropanol‐acetonitrile containing 0.1% formic acid and 10 mM ammonium formate (B2). Chromatographic separation was achieved using a 2 μL injection volume with a flow rate maintained at 0.25 mL/min.

#### 2.5.2 Mass Spectrometric Parameters

ESI‐MSn analyses were conducted with spray voltages of 3.5 kV (positive mode) and 2.5 kV (negative mode) for positive and negative modes, respectively. Gas flow parameters included sheath and auxiliary gases maintained at 30 and 10 arbitrary units, respectively. The capillary heating system operated at 325°C throughout the experiments. Mass spectral acquisition employed an orbitrap detector scanning across the 150–2000 m/z range with full‐scan resolution set to 35,000. For structural characterization, data‐dependent MS/MS analysis was executed using higher energy collisional dissociation (HCD) with collision energy normalized to 30 eV.

The MS/MS spectra underwent data refinement through application of dynamic exclusion criteria to eliminate extraneous information.

### 2.6 ECG‐EVLP Dye Marker Method

A 1‐μL aliquot of PKH26 fluorescent dye (Merck KGaA, Darmstadt, Germany) was introduced into ECG‐EVLP suspension (200 μg/mL concentration) at 5 μM working concentration. The mixture was maintained at 37°C for 60 min before removing unbound dye through ultracentrifugation at 120,000 *g* for 30 min. The ultracentrifuge rotor type was SW‐70 Ti. The pelleted material was subsequently reconstituted in PBS solution following centrifugation.

### 2.7 In Vitro Cellular Uptake Assay of ECG‐EVLP

HaCat cells and L929 cells were plated at a density of 10,000 cells per well in 12‐well plates containing circular coverslips, followed by a 24‐h incubation period at 37°C. Subsequently, the cultures were exposed to PKH26‐labeled ECG‐EVLP suspension (200 μg/mL) in fresh medium for 12 h under standard culture conditions. Post‐staining with Hoechst and fluorescein isothiocyanate (FITC) fluorescent markers, the circular coverslips were carefully extracted from the culture plates, inverted, and mounted on slides for subsequent analysis. Hoechst was used to label the nucleus, and FITC was used to label the cytoskeleton.

Following sealing, the slides underwent imaging using a laser confocal microscope (Leica stellaris 5, Germany).

### 2.8 Cell Culture

Human keratinocyte (HaCat) and NCTC clone 929 (L929) cell lines were obtained from the American Type Culture Collection (ATCC), with cell line authenticity verified through short tandem repeat (STR) profiling. These cells were maintained in Dulbecco's modified Eagle medium (DMEM) (GIBCO, Fisher Scientific, Italy) supplemented with 10% fetal bovine serum (FBS) (GIBCO, Fisher Scientific, Italy) and 1% penicillin–streptomycin antibiotic mixture, cultured under standard conditions of 37°C with a 5% CO_2_ atmosphere.

### 2.9 Cell Counting Kit‐8 (CCK‐8) Assay

HaCat cells and L929 cell suspensions (3 × 10^3^ cells/well) were plated into 96‐well culture plates. Following a 24‐h incubation period, the cultures were exposed to ECG‐EVLP suspensions at three particle concentrations (150, 300, and 450 μg/mL) and maintained under standard culture conditions (37°C, 5% CO_2_) for 24–48 h. Parallel control groups received equivalent volumes of PBS. Posttreatment procedures involved triple PBS rinsing followed by the addition of DMEM supplemented with 10% detection reagent. After 2 h of incubation under physiological conditions, optical density measurements at 450 nm were recorded using a microplate detection system. The experiments described above were conducted in three biological replicates.

### 2.10 Cell Scratch Assay

HaCat cells and L929 cells were grown in 6‐well plates until reaching 80% cellular density. Uniform scratches were generated using a sterile 1000 μL pipette tip, followed by two PBS washes to eliminate detached cellular material. Each well received 1500 μL of DMEM supplemented with 2% FBS before documenting the initial wound area through microscopic imaging at baseline (0 h).

Following initial setup, all experimental groups except the control (maintained in 1500 μL of 2% FBS‐supplemented DMEM) received modified media containing 2% FBS with ECG‐EVLP concentrations of 150 and 300 μg/mL. Cellular cultures were subsequently maintained for 48 h under standard incubation conditions (37°C, 5% CO_2_ atmosphere).

Wound closure was evaluated utilizing an inverted microscope (Olympus, Japan), with standardized photographic documentation of lesion sites maintained at identical positions across observation intervals (0, 24, and 48 h). ImageJ software facilitated quantitative analysis through comparative measurement of wound dimensions at specified time points.

### 2.11 Transwell Assay

L929 cells were plated into 24‐well culture plates and allowed to adhere overnight. Following a PBS wash, the cells were maintained for 24 h in serum‐deprived DMEM supplemented with ECG‐EVLP at concentrations of 150 and 300 μg/mL. After treatment, the cells were harvested and transferred to the upper compartment of transwell inserts within a 24‐well plate. The migration assay was conducted using transwell chambers, with the lower compartment containing DMEM enriched with 10% fetal bovine serum. The pore size of the transwell membrane was 8.0 μm.

L929 cells underwent additional 24‐h incubation before stationary cells were eliminated using cotton swabs. Migratory populations were subsequently immobilized in 4% paraformaldehyde solution and subjected to 0.1% crystal violet coloration.

### 2.12 Reactive Oxygen Species Detection

Intracellular reactive oxygen species (ROS) concentrations were quantified employing the Reactive Oxygen Species Detection Kit (BL714A; Biosharp, Guangzhou, China). HaCat cells and L929 cells were plated in 6‐well culture dishes with an initial seeding density of 5 × 10^5^ cells per well. Following group‐specific treatments administered over a 3‐h period, hydrogen peroxide (H_2_O_2_) which was used as a positive control reagent was introduced to the cellular cultures. Subsequent incubation was conducted under light‐protected conditions at 37°C for 60 min to induce ROS production.

Following centrifugation, cells were harvested and treated with a diluted fluorescent probe solution containing diacetyl dichlorofluorescein (DCFH‐DA). The mixture underwent 30‐min incubation at 37°C under light‐protected conditions, followed by two washes using serum‐free culture medium. Fluorescence signals were visualized microscopically, while quantitative analysis of cellular mean fluorescence intensity (MFI) was performed through flow cytometry (the number of injected cells was set to 5000). The excitation wavelength was 450–490 nm, and the emission wavelength was 520–530. The output parameter MFI, the average fluorescence intensity per cell, was used to represent cellular ROS levels.

### 2.13 Wound Healing Effects of ECG‐EVLP

Male C57BL/6 mice (SPF, aged 6–8 weeks) were obtained from Guangdong Medical Laboratory Animal Center. Following arrival, the animals underwent a 7‐day acclimatization period in standardized laboratory conditions with continuous access to food and water. These subjects were collectively housed in stainless steel enclosures containing three individuals per cage, maintained under controlled environmental parameters including a 12‐h photoperiod regimen.

Anesthesia induction was achieved through 4% isoflurane vapor administration, with subsequent maintenance using 2% isoflurane. The dorsal surgical field underwent depilation and antiseptic preparation. Under aseptic protocol, a circular full‐thickness cutaneous defect measuring approximately 10 mm in diameter was created along the dorsal midline. Experimental cohorts received four subcutaneous injections at 3, 6, 9, and 12 o'clock positions around per wound, each administering 100 μL of therapeutic solution containing 1 mg/mL.

The mice were randomly assigned to two experimental cohorts: the PBS control group received equivalent volumes of phosphate‐buffered saline through subcutaneous injection on Days 0, 3, and 7, whereas the ECG‐EVLP treatment group was administered the ECG‐EVLP solution according to the same schedule.

Quantitative analysis of wound areas was performed using ImageJ software at predetermined intervals (Days 0, 3, 7, 11, and 14 posttreatment), with subsequent calculation of wound closure rates. Following the 14‐day observation period, subjects underwent euthanasia for comprehensive biological sample collection, including both dermal tissues and systemic circulation specimens.

### 2.14 Histological Analysis

Tissue samples underwent fixation in 10% neutral buffered formalin followed by paraffin embedding. Using a microtome, 5‐μm‐thick sections were prepared from the paraffin blocks. Sequential processing included xylene‐based dewaxing and gradual rehydration through an alcohol gradient. Histological staining was performed using both hematoxylin–eosin (HE) for cellular morphology and Masson's trichrome for collagen visualization. Prepared slides were examined using light microscopy with digital image acquisition. ImageJ software was used to analyze the visual field images (100×) to evaluate the repair of wound skin tissue.

### 2.15 Enzyme‐Linked Immunosorbent Assay (ELISA)

Mouse peripheral blood samples were gathered in additive‐free collection tubes and maintained at ambient temperature for 60 min to facilitate clot formation. After complete blood coagulation and subsequent serum separation through natural sedimentation, the supernatant was isolated using refrigerated centrifugation (1500 *g*, 10 min at 4°C). Analytical operations were conducted following manufacturer‐provided protocols from commercial assay kits. Serum concentrations of inflammatory mediators TNF‐α, IL‐6, and IL‐1β were quantified using ELISA kits supplied by Servicebio Biotechnology Co. Ltd.

### 2.16 Western Blot Analysis

HaCat cells and L929 cells were plated into six‐well plates with an initial density of 1.2 × 10^5^ cells per well and maintained in culture for 24 h. ECG‐EVLP was administered at varying concentrations (150, 300, and 450 μg/mL) to the experimental groups. Following a 48‐h incubation period, the cells were harvested. A lysis cocktail containing RIPA buffer mixed with protease and phosphatase inhibitors in a 100:1:1 ratio was applied to isolate cellular proteins. The resulting lysates underwent subsequent processing for protein extraction and quantification.

Protein concentrations were quantified and standardized by BCA to ensure uniformity.

Protein samples were resolved using 10% sodium dodecyl sulfate‐polyacrylamide gel electrophoresis (SDS‐PAGE) and electrophoretically transferred onto PVDF membranes from Merck KGaA (Darmstadt, Germany). Following transfer, the blots were treated with blocking buffer to reduce background interference prior to overnight exposure to primary antibodies at 4°C.

Subsequently, the membranes were exposed to secondary antibodies at 25°C following multiple washes with Tween 20‐supplemented tris‐buffered saline (TBST). For detection, protein bands were visualized through an ultrasensitive enhanced chemiluminescence (ECL) detection platform, with subsequent densitometric analysis performed using ImageJ software (version 1.8.0).

The following primary antibodies were used in this study: anti‐Phospho‐AKT (1:1000, 80455‐1‐RR; Proteintech Group, Wuhan, China), anti‐AKT (1:1000, 10176‐2‐AP; Proteintech Group), anti‐VEGFA (1:1000, 19003‐1‐AP; Proteintech Group), anti‐Beta Tubulin (1:2000, 10094‐1‐AP; Proteintech Group), anti‐GAPDH (1:10,000, ab181602; Abcam, UK), anti‐Bcl‐2 (1:2000, ab182858; Abcam), anti‐Bax (1:2000, ab32503; Abcam).

### 2.17 Statistical Analysis

Data are expressed as mean values ± standard deviation. Statistical analyses were conducted using IBM SPSS Statistics 27.0. Comparisons among three or more groups were analyzed through one‐way ANOVA followed by Tukey's post hoc testing, whereas differences between two groups were examined using two‐tailed Student's *t*‐tests. Statistical significance was defined as *p < 0.05, **p < 0.01, and ***p < 0.001, demonstrating the statistical significance thresholds corresponding to the detected variations.

Additional experimental details are provided in the Supporting Information.

## 3 Result

### 3.1 Isolation and Morphological Characterization of ECG‐EVLP

**FIGURE 3:**
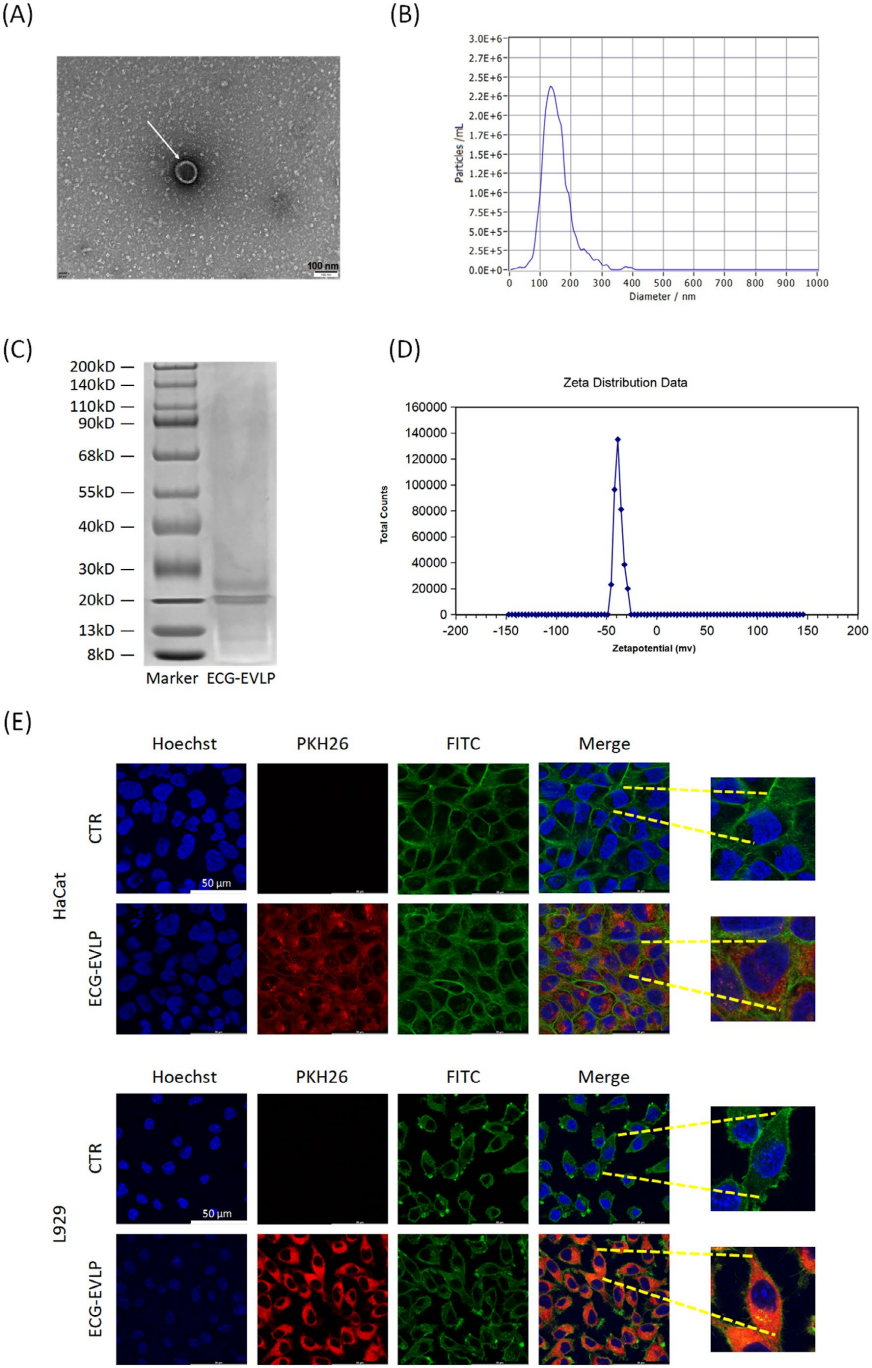
Morphological characterization, protein Coomassie brilliant blue staining, and cellular uptake assay of ECG‐EVLP. (A) Transmission electron microscopy (TEM), ECG‐EVLP indicated by white arrow. (B) NTA detection. (C) Coomassie brilliant blue staining. (D) Zeta potential. (E) ECG‐EVLP was taken up by HaCat cells and L929 cells.

ECG‐EVLP was isolated and purified from ECG juice by polyethylene glycol precipitation. The isolated ECG‐EVLP was then detected by TEM, NTA, and zeta potential analysis. The results showed that the isolated particles had a typical saucer‐like morphology (Figure 3A). Additionally, they were nanoscale particles with a diameter distribution peaking at 135 nm (Figure 3B). The NTA results demonstrated that the particle size distribution exhibited a single peak at 135 nm, confirming monodispersity of ECG‐EVLP and suggesting the structural integrity of the ECG‐EVLP. Zeta potential analysis showed that ECG‐EVLP had a negative zeta potential and zeta potential peak was −38.7 mV (Figure 3D). Zeta potential is a core parameter to characterize the stability of the colloidal system, and an absolute value greater than 30 mV indicates that the system is stable and ECG‐EVLP can be uniformly and stably dispersed in this solution system. Analysis of protein size in ECG‐EVLP by SDS‐PAGE and Coomassie brilliant blue staining showed that the protein molecular weight of ECG‐EVLP was mainly distributed in the range of 20 to 30 kDa (Figure 3C).

### 3.2 Untargeted Metabolomics and Lipidomics

**FIGURE 4:**
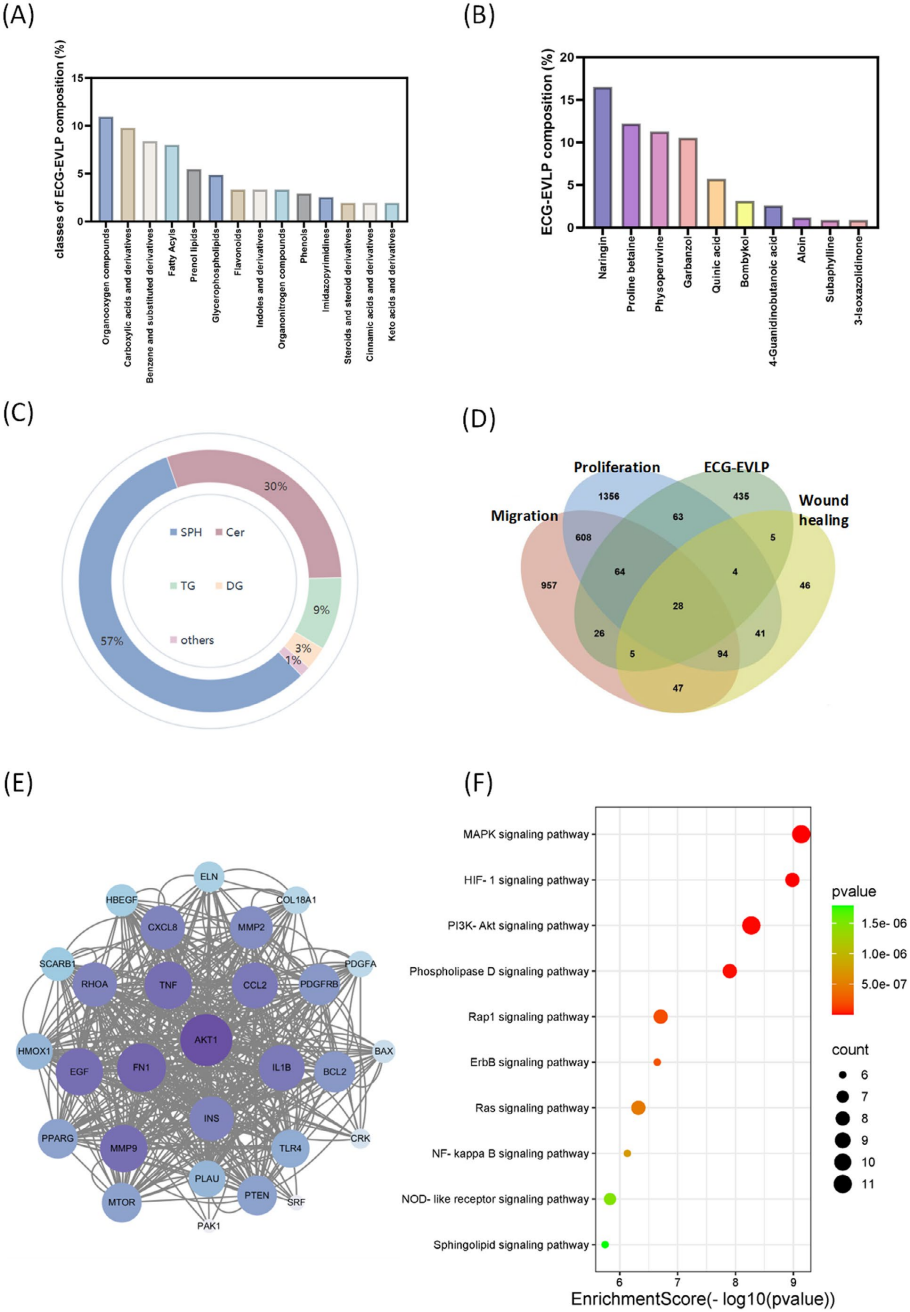
Component characterization of ECG‐EVLP. (A) Classes of major compounds in ECG‐EVLP detected by non‐targeted metabolomics. (B) The major compound components (except lipid components) in ECG‐EVLP detected by untargeted metabolomics. (C) The major lipid components in ECG‐EVLP detected by untargeted lipidomics. (D) Venn diagram of gene targets for active components of ECG‐EVLP and proliferation, migration, and wound healing. (E) PPI network diagram of the 28 intersection genes. (F) KEGG enrichment analysis.

The results of untargeted metabolomics showed that the main classes of compounds in ECG‐EVLP were organooxygen compounds, carboxylic acids and derivatives, benzene and substituted derivatives, fatty acyls, and prenol lipids. Among these, naringin, proline betaine, physoperuvine, and garbanzol were more abundant in the non‐lipid components (Figure 4A,B). Lipidomics results showed that sphingosine was the major lipid component in ECG‐EVLP (Figure 4C).

Subsequently, five main active compounds (sphingosine, naringin, proline betaine, physoperuvine, and garbanzol) in ECG‐EVLP were selected for subsequent analysis by mining and comparing the Genecard database and relevant literature. By searching in Genecard (https://www.genecards.org/), we learned that these active compounds involved 630 predicted gene targets, of which 28 potential gene targets intersect between the regulatory networks of cell proliferation, migration, and wound healing (Figure 4D). Enrichment analysis of these 28 genes was performed using Kyoto Encyclopedia of Genes and Genomes (KEGG) databases to find out the pathways that might be involved (Figure 4F). The results showed that the active components of ECG‐EVLP were more significantly enriched in PI3K/AKT and VEGF signaling pathways (Figure 4F). Meanwhile, the PPI network verified that AKT was a potential target for ECG‐EVLP to promote skin wound healing (Figure 4E).

### 3.3 ECG‐EVLP Promotes Cell Proliferation and Migration of HaCat Cells and L929 Cells In Vitro

**FIGURE 5:**
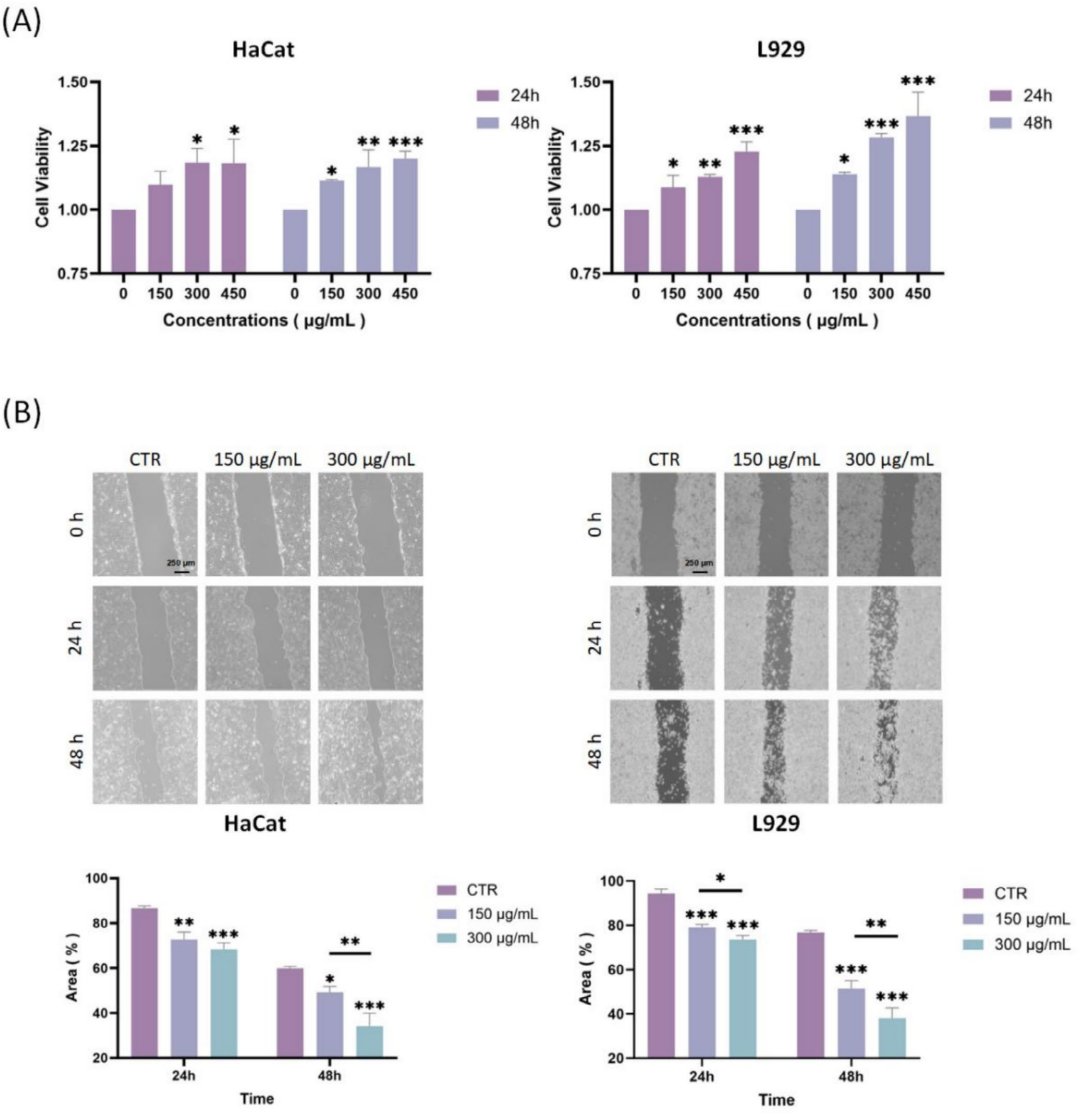
Effect of ECG‐EVLP on proliferation and migration of HaCat cells and L929 cells. (A) Proliferation of HaCat cells and L929 cells treated with ECG‐EVLP was measured by CCK‐8 assay. (B) Migration of HaCat cells and L929 cells treated with ECG‐EVLP was assessed by a wound healing assay. Data are from one experiment representative of three independent experiments and are presented as mean ± SD (*n* = 3 technical replicates). Statistical significance was determined by one‐way ANOVA followed by Tukey's post hoc test. *p < 0.05, **p < 0.01, ***p < 0.001.

To investigate the effect of ECG‐EVLP on the proliferation of skin cells, the proliferation ability of ECG‐EVLP was detected by HaCat cells and L929 cells. Cellular uptake assay was used to determine whether ECG‐EVLP could accumulate in skin cells. Confocal microscopy of HaCat cells and L929 cells incubated with PKH26‐labeled ECG‐EVLP showed that ECG‐EVLP was taken up by both cell lines (Figure 3E). Firstly, CCK8 assay was performed to detect the toxic effect of ECG‐EVLP on HaCat cells and L929 cells by incubating cells with different concentrations of ECG‐EVLP. The results showed that the proliferation of HaCat cells induced by ECG‐EVLP was increased at 150 and 300 μg/mL concentrations after 24 h and at 150, 300, and 450 μg/mL concentrations after 48 h, while the proliferation of L929 cells was significantly increased at 150, 300, and 450 μg/mL concentrations after both 24 and 48 h (Figure 5A).

**FIGURE 6:**
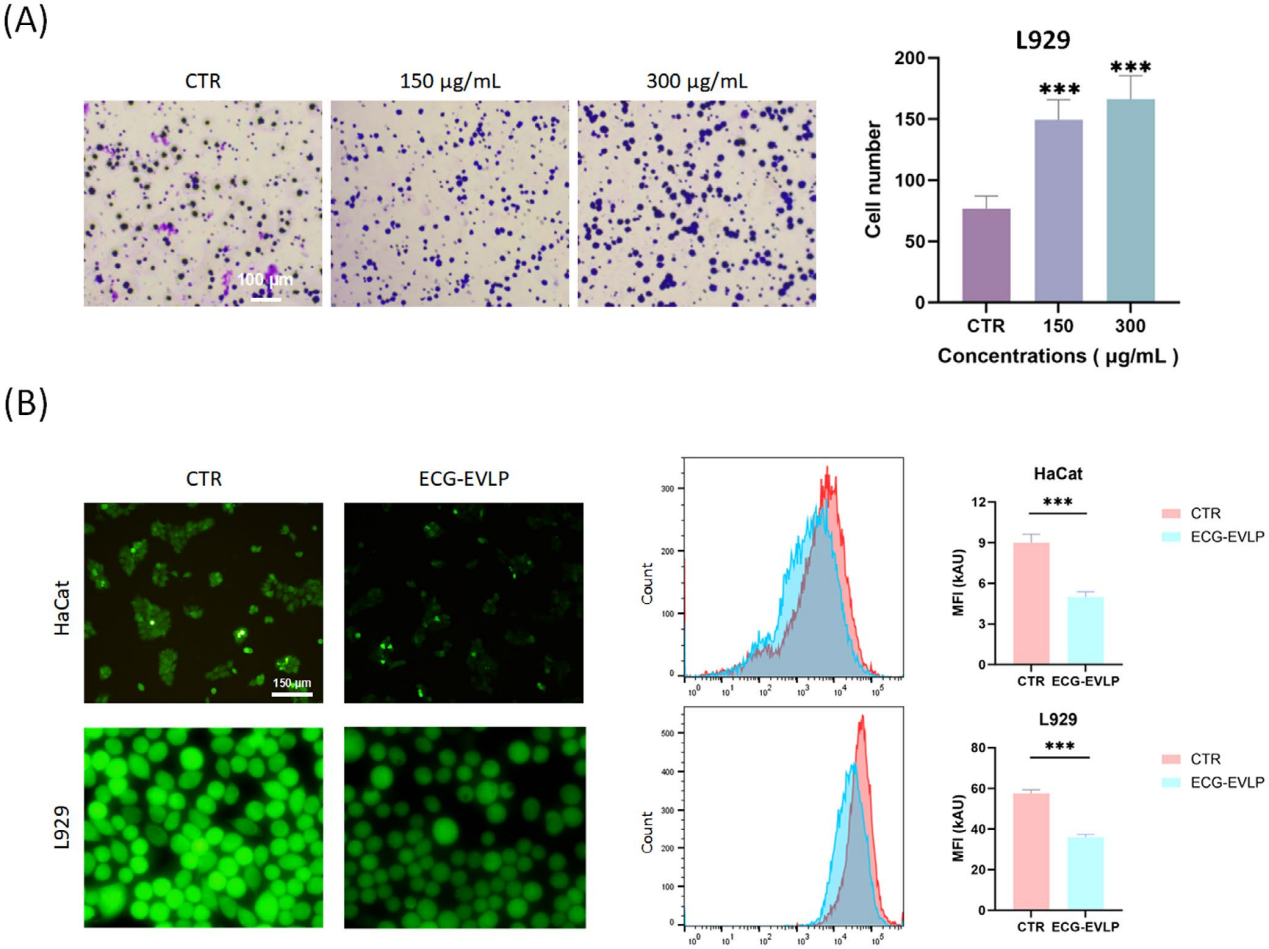
Effect of ECG‐EVLP on migration of L929 cells and on the ROS level of HaCat cells and L929 cells. (A) Migration of L929 cells after treatment with ECG‐EVLP was assessed by a transwell assay. Statistical significance was determined by one‐way ANOVA followed by Tukey's post hoc test. (B) Intracellular ROS levels in HaCat cells and L929 cells after treatment with ECG‐EVLP were measured. Data are from one experiment representative of three independent experiments and are presented as mean ± SD (*n* = 3 technical replicates). Statistical significance was determined by Student's *t*‐test. ***p < 0.001.

Transwell assay and cell scratch assay were used to confirm the effect of ECG‐EVLP on cell migration. In the scratch assay (Figure 5B), different concentrations of ECG‐EVLP significantly reduced the scratch area of HaCat cells and L929 cells after 24 and 48 h compared with the control group. In the transwell assay (Figure 6A), different concentrations of ECG‐EVLP significantly increased the number of migrated L929 cells after 24 h compared with the control group.

### 3.4 Effect of ECG‐EVLP on the ROS Level of HaCat Cells and L929 Cells In Vitro

To evaluate the antioxidant activity of ECG‐EVLP, we employed the DCFH‐DA probe in an in vitro setting. All cells were pretreated with a positive control (H_2_O_2_) to stimulate reactive oxygen species (ROS) production. Fluorescence intensity was quantified using both inverted microscopy and flow cytometry (Figure 6B). Compared to the control group, the fluorescence intensity in the ECG‐EVLP group was significantly reduced. This indicates that cellular uptake of ECG‐EVLP partially mitigated the overproduction of ROS induced by the positive control.

### 3.5 Effect of ECG‐EVLP on Protein Expression in HaCat Cells and L929 Cells

**FIGURE 7:**
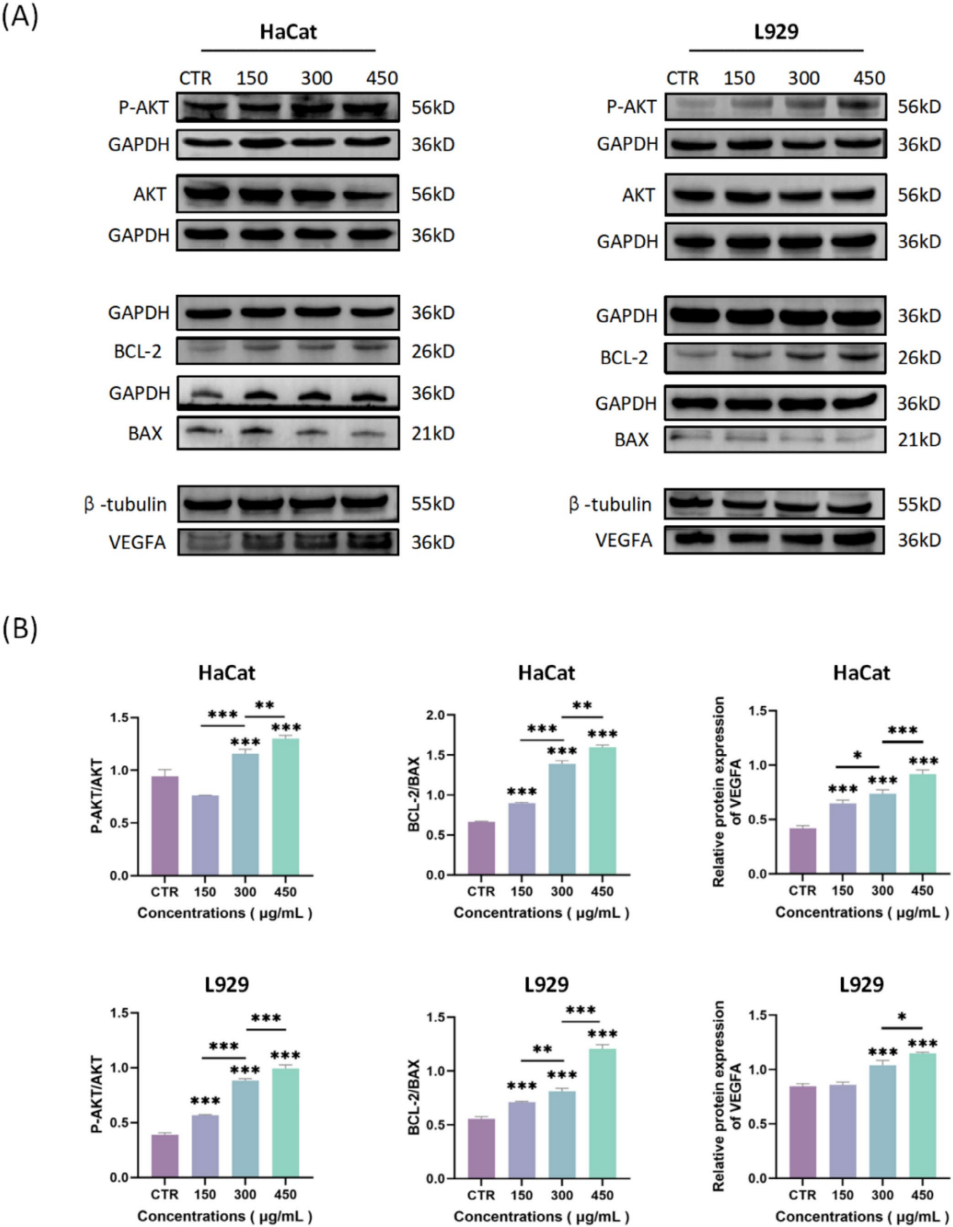
Effect of ECG‐EVLP on protein expression in HaCat cells and L929 cells. (A) Representative Western blots showing the expression of P‐AKT, AKT, BCL‐2, BAX, and VEGFA in HaCat cells (left) and L929 cells (right) after treatment with ECG‐EVLP. (B) Quantification of protein band intensities and the ratios of P‐AKT/AKT and BCL‐2/BAX. Data are from one experiment representative of three independent experiments and are presented as mean ± SD (*n* = 3 technical replicates). Statistical significance for all panels was determined by one‐way ANOVA followed by Tukey's post hoc test. *p < 0.05, **p < 0.01, ***p < 0.001.

To further investigate the effects of ECG‐EVLP on skin cells and to verify our hypothesis that these vesicles may modulate both the VEGF/AKT pathway and the mitochondrial apoptotic pathway, we examined the expression levels of key regulatory proteins using Western blot analysis. Compared with the control group, ECG‐EVLP increased the expression of P‐AKT and the ratio of P‐AKT/AKT in HaCat cells and L929 cells in a concentration‐dependent manner. ECG‐EVLP also increased the expression of BCL‐2 and decreased the expression of BAX in HaCat cells and L929 cells in a concentration‐dependent manner, resulting in an increase in BCL‐2/BAX ratio by upregulating BCL‐2 and downregulating BAX (Figure 7A,B). Moreover, ECG‐EVLP stimulation also increased the expression level of VEGFA in HaCat cells and L929 cells (Figure 7A,B). For the detection of VEGFA, β‐tubulin was used as an internal control; for all other targets, GAPDH was used.

### 3.6 ECG‐EVLP Accelerates Skin Wound Healing of C57BL/6 Mice In Vivo

**FIGURE 8:**
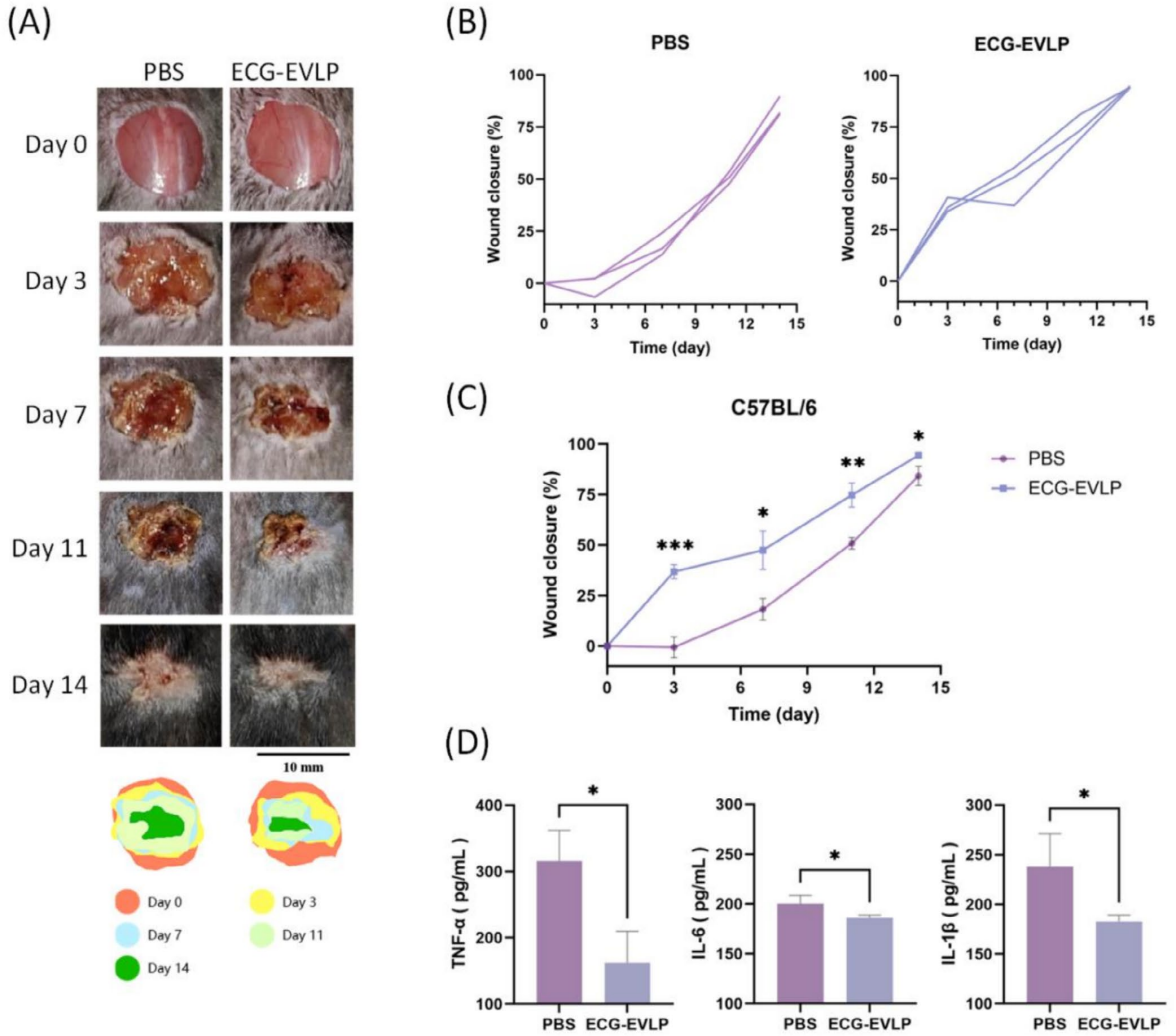
Effect of ECG‐EVLP on skin wound healing and serum inflammatory factors in C57BL/6 mice. (A) Representative photographs of wounds from C57BL/6 mice on Days 0, 3, 7, 11, and 14. (B) Wound closure rate over time. (C) Comparison of wound closure rate between the ECG‐EVLP and PBS groups at each indicated time point. (D) Serum levels of TNF‐α, IL‐6, and IL‐1β on Day 14. For (C) and (D), data are presented as mean ± SD (*n* = 3 biologically independent mice per group). All significance markers indicate comparisons between the ECG‐EVLP group and the PBS control group. Statistical significance for between‐group comparisons in (C) and (D) was determined using Student's *t*‐test. *p < 0.05, **p < 0.01, ***p < 0.001.

To evaluate the therapeutic potential of ECG‐EVLP in skin wound healing, we performed in vivo experiments using C57BL/6 mouse skin wound models. The results showed that the skin wound healing rate of ECG‐EVLP treated mice was significantly enhanced. During the 14‐day observation period, wound healing was significantly improved in the ECG‐EVLP group compared with the control group on each assessed day: Day 3 (***p < 0.001 vs. control), Day 7 (*p < 0.05 vs. control), Day 11 (**p < 0.01 vs. control), and Day 14 (*p < 0.05 vs. control) (Figure 8A–C).

### 3.7 Effects of ECG‐EVLP on TNF‐α, IL‐6, and IL‐1β in Serum of C57BL/6 Mice

On Day 14 of in vivo experiments, the expressions of pro‐inflammatory factors (TNF‐α, IL‐6, and IL‐1β) in the serum of mice were accessed by ELISA. The results indicated that ECG‐EVLP treatment effectively reduced the overexpression of TNF‐α, IL‐6, and IL‐1β in the model mice (Figure 8D).

### 3.8 Effect of ECG‐EVLP on Tissue Levels During Skin Wound Healing in C57BL/6 Mice

**FIGURE 9:**
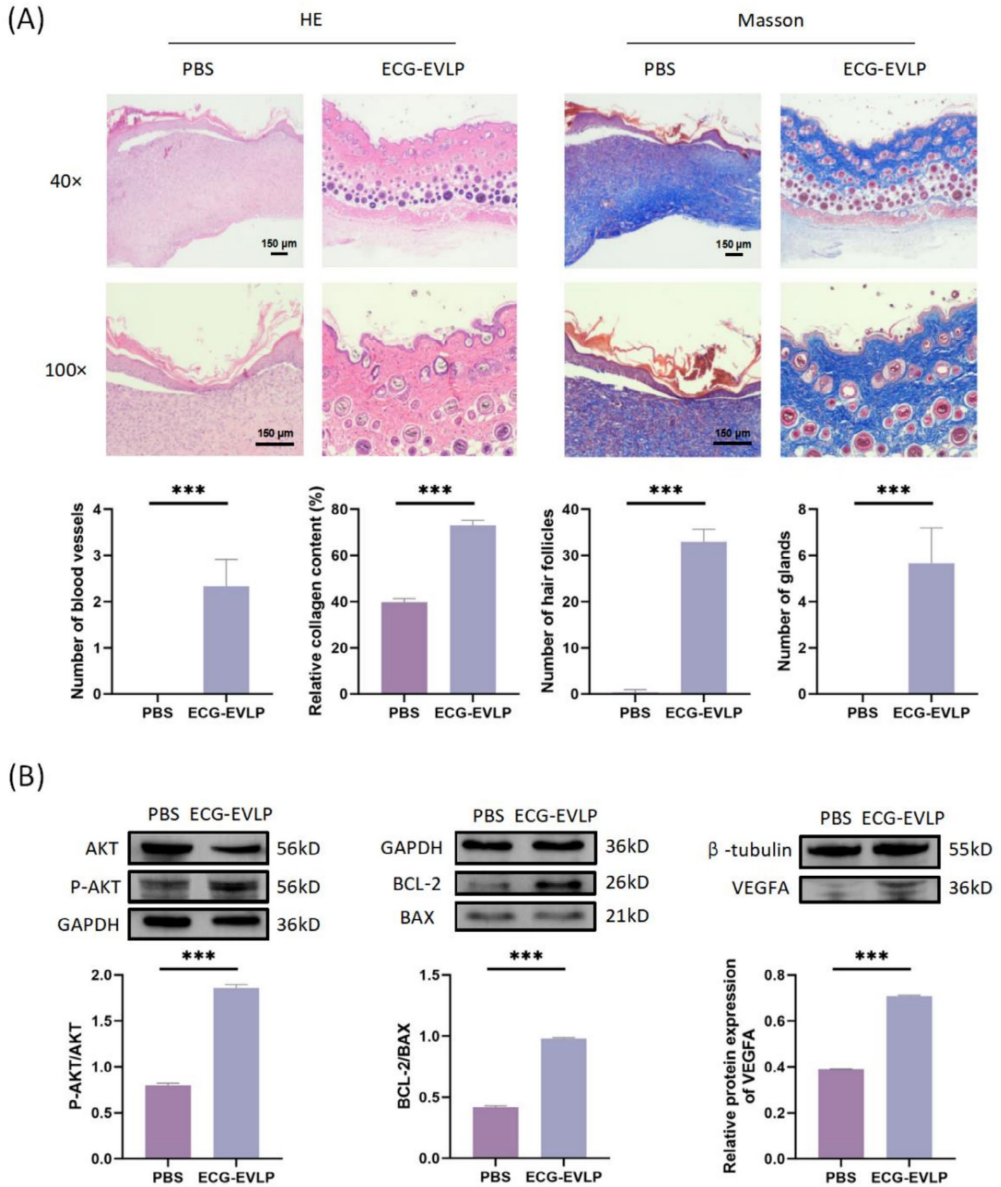
Effect of ECG‐EVLP on histology and protein expression in skin wound healing of C57BL/6 mice. (A) Upper panel: Representative H&E and Masson's trichrome staining of wound tissues from C57BL/6 mice at Day 14. Lower panel: Quantitative evaluation of skin repair parameters. (B) Western blot analysis of key signaling molecules in wound tissues. Left: P‐AKT/AKT ratio. Middle: BCL‐2/BAX ratio. Right: VEGFA expression level. All quantitative data are presented as mean ± SD (*n* = 3 biologically independent mice per group). Protein band intensities were quantified, and the respective ratios were calculated. Statistical significance for between‐group comparisons was determined by Student's *t*‐test. ***p < 0.001.

Skin wound tissue of C57BL/6 mice was stained with HE and Masson on Day 14. At Day 14, sections of regenerated skin tissue treated with ECG‐EVLP showed a significant increase in new blood vessels and collagen production. In addition, abundant skin appendages, including hair follicles and glands, were observed in wounds treated with ECG‐EVLP (Figure 9A). These findings provide evidence that ECG‐EVLP promotes the restoration of skin tissue structure.

### 3.9 Effect of ECG‐EVLP on Protein Expression Levels in Skin Wound Tissue of C57BL/6 Mice

According to the results of Western blot, ECG‐EVLP upregulated the expression of P‐AKT and unchanged total AKT remains in the wound tissue of C57BL/6 mice, resulting in an increase in the ratio of P‐AKT/AKT in the wound tissue. At the same time, ECG‐EVLP upregulated the expression of BCL‐2 and downregulated the expression of BAX, resulting in the increase of BCL‐2/BAX by upregulating BCL‐2 and downregulating BAX (Figure 9B). Not only that, ECG‐EVLP stimulation also increased VEGFA expression in the skin wound tissue of C57BL/6 mice (Figure 9B).

## 4 Discussion

As the largest organ of the body, the skin serves as the primary barrier against the external environment. Skin wounds, however, are common and can lead to various complications such as bacterial infections and ulcer formation. While conventional hormone‐based therapies can be effective under certain conditions, they are often associated with adverse effects and a risk of drug resistance. Owing to their superior biocompatibility and therapeutic efficacy, biomimetic therapies have thus gained increasing attention as promising alternatives in clinical practice. Although the use of P‐EVLP in wound healing was initially met with skepticism, accumulating evidence indicates their ability to modulate disease processes in animal models, and a number of in vitro studies have supported their therapeutic potential (Feng et al. 2023). P‐EVLP derived from sources such as wheat (Şahin et al. 2019), grapefruit (Savcı et al. 2021), *Aloe vera* (Kim and Park 2022; Ramírez et al. 2024), tomato (Daniello et al. 2024), *Morus* sp. (mulberry) (Garrett et al. 2024), *Physalis peruviana* (Natania et al. 2025), *Baeckea frutescens* L. (Safwan Kamarazaman et al. 2024), *Opuntia ficus‐indica* (Valentino et al. 2024), and Dendrobium (Tu et al. 2024) have been investigated for wound healing applications. Compared with these previously reported P‐EVLP, ECG‐EVLP exhibits a unique metabolomic profile enriched in sphingosine and naringin, both of which have been independently linked to enhanced wound repair (Kawanabe et al. 2007; Yen et al. 2022). Moreover, while some other P‐EVLP have been shown to promote cell migration and proliferation, our study provides mechanistic evidence that ECG‐EVLP specifically activates the VEGF/AKT pathway and suppresses mitochondrial apoptosis, offering deeper insight into its pro‐healing effects.

The AKT signaling pathway plays a critical role in regulating essential cellular processes, including proliferation, apoptosis, migration, and angiogenesis—a notion supported by numerous studies (Chen et al. 2020; Xiu et al. 2022). For instance, antioxidant‐engineered milk‐derived extracellular vesicles (MEVs) have been shown to accelerate wound healing by activating the AKT‐mediated VEGF signaling pathway (Fan et al. 2023). Other reports further underscore the importance of both the AKT and VEGF pathways in epidermal cell proliferation, migration, and angiogenesis during wound healing (Li et al. 2023; Zhang, Zouboulis, and Xiao 2024). Mechanistically, VEGFA binding to vascular endothelial growth factor receptor 2 (VEGFR2) recruits phosphoinositide 3‐kinase (PI3K), leading to its autophosphorylation. Activated PI3K then phosphorylates AKT, which in turn stimulates downstream effectors such as BRCA1, eNOS, and BCL‐2, collectively promoting cell proliferation, migration, and angiogenesis, while also inhibiting apoptosis. Apoptosis is regulated by the BCL‐2 family through mitochondrial permeability. The antiapoptotic protein BCL‐2 localizes to the mitochondrial outer membrane and prevents cytochrome C release. In contrast, BAX—a proapoptotic member of the BCL‐2 family—resides in the cytosol and translocates to mitochondria upon apoptotic stimulation, triggering cytochrome C release. Cytosolic cytochrome C activates Caspase‐3, initiating apoptosis. BCL‐2 binds directly to BAX, inhibiting its oligomerization and pore formation on the mitochondrial membrane, thereby preserving membrane integrity. This interaction prevents the leakage of cytochrome C and other apoptotic factors into the cytoplasm, blunts Caspase cascade activation, and ultimately suppresses apoptosis. Therefore, the BCL‐2/BAX ratio is a critical determinant of cellular fate (Ficai et al. 2025). Together, these findings illustrate the intricate relationship between AKT signaling and apoptosis regulation, highlighting the central role of the AKT pathway in cellular activities relevant to wound healing and angiogenesis.

Untargeted metabolomic and lipidomic analyses of ECG‐EVLP identified five major active components. Among these, sphingosine (Chen et al. 2023; Kawanabe et al. 2007; Tang et al. 2023) and naringin (Yen et al. 2022) have been previously confirmed to promote wound healing, suggesting that these compounds may constitute key active components of ECG‐EVLP responsible for its regenerative properties.

Cellular uptake assays confirmed that ECG‐EVLP is efficiently internalized by both HaCat cells and L929 cells, enabling subsequent phenotypic investigations. CCK‐8 assays revealed that ECG‐EVLP stimulated the proliferation of HaCat cells and L929 cells. Cell scratch assays and transwell assays further demonstrated that ECG‐EVLP significantly enhanced the migration of both cell types. As cell migration is essential for skin wound healing—enabling cells to relocate to the wound site and reconstruct tissue—it represents a critical process in accelerating tissue regeneration. In vivo experiments using a mouse wound healing model confirmed that ECG‐EVLP significantly improved the wound closure rate from the third day onward. An important hallmark of effective healing is a shortened inflammatory phase and prevention of wound infection. Consistent with this, after 14 days of treatment, ECG‐EVLP markedly reduced serum levels of TNF‐α, IL‐6, and IL‐1β in model mice, suggesting a more rapid resolution of inflammation. Given that excessive ROS production is known to impair wound healing (Dong et al. 2025; Lu et al. 2024), we also evaluated the antioxidant capacity of ECG‐EVLP. Our in vitro results showed that ECG‐EVLP partially counteracted ROS overproduction induced by a positive control, indicating that ROS attenuation may be one mechanism through which ECG‐EVLP facilitates wound recovery.

Multiple studies have emphasized the importance of the AKT and VEGF signaling pathways, as well as the mitochondrial apoptosis pathway, in wound healing. We hypothesized that ECG‐EVLP promotes AKT phosphorylation via VEGFA upregulation, thereby enhancing proliferation, migration, and angiogenesis. Concurrently, ECG‐EVLP reduced BAX expression, while P‐AKT enhanced BCL‐2 expression, collectively attenuating apoptosis. Western blot analysis confirmed that ECG‐EVLP increased VEGFA expression and elevated the P‐AKT/AKT ratio in HaCat cells, L929 cells, and mouse wound tissues. These results suggest that ECG‐EVLP activates the VEGF/AKT pathway to promote skin wound healing. Moreover, ECG‐EVLP upregulates BCL‐2 and downregulates BAX in all tested models, indicating that it modulates mitochondrial permeability and reduces apoptosis via the BCL‐2/BAX axis, further supporting skin wound regeneration.

While these findings offer valuable mechanistic insights, this study is limited by the lack of direct comparison between formulated ECG‐EVLP and non‐formulated ECG extracts. Future studies should systematically evaluate bioavailability, pharmacokinetics, and in vivo stability to clarify the advantages of ECG‐EVLP formulation. This work not only supports the therapeutic potential of ECG‐EVLP as a natural nanocarrier for tissue repair but also broadens the understanding of how P‐EVLP contribute to regenerative medicine. Further research is warranted to explore their clinical translation and compare their efficacy with other plant‐derived and synthetic vesicle systems.

## 5 Conclusion

In summary, our study demonstrates that ECG‐EVLP significantly accelerates skin wound healing in mice by promoting cell proliferation, reducing apoptosis, enhancing migration and collagen production at the wound site. Specifically, in C57BL/6 mice, the ECG‐EVLP group exhibited a higher healing rate and demonstrated anti‐inflammatory properties. In vitro experiments with HaCat cells and L929 cells further support the hypothesis that the therapeutic potential of ECG‐EVLP may be attributed to its ability to promote the proliferation and migration of keratinocytes and fibroblasts. In addition, ECG‐EVLP reduced the levels of inflammatory factors in the model mice and partially abolished the ROS produced by HaCat cells and L929 cells induced by positive control. The underlying mechanisms may involve the activation of the VEGF/AKT pathways, as well as the suppression of the mitochondrial apoptosis pathway in response to ECG‐EVLP stimulation.

However, a notable limitation of the current study is the lack of a comprehensive dose–response assessment in vivo, which is still to be improved. Future studies will focus on establishing a detailed dose–response relationship and identifying the minimum effective dose to optimize the therapeutic window of ECG‐EVLP. Looking ahead, given the convenience, biocompatibility, and the potential for further optimization through nanoparticle engineering or innovative delivery systems, ECG‐EVLP holds considerable promise for clinical applications beyond skin wound healing, extending to diabetic wounds and burns. We envision that ECG‐EVLP represents a promising therapeutic approach to enhance skin wound healing in patients in the future.

## Author Contributions

**Yingjie Xiong:** conceptualization (equal), data curation (equal), formal analysis (equal), visualization (equal), writing – original draft (equal). **Zanxiang Luo:** formal analysis (equal), methodology (equal), software (equal). **Jingxiu Zhao:** investigation (equal), resources (equal), validation (equal), visualization (equal). **Chengshi Fu:** formal analysis (equal), methodology (equal), software (equal). **Yujia Song:** software (equal), validation (equal), visualization (equal). **Jinyong He:** formal analysis (equal), methodology (equal), software (equal). **Jiahui Gao:** data curation (equal), methodology (equal), resources (equal), software (equal). **Zejie Su:** formal analysis (equal), investigation (equal), methodology (equal), software (equal). **Lie Liu:** validation (equal), visualization (equal). **Xiangyun Teng:** funding acquisition (equal), investigation (equal), project administration (equal), writing – review and editing (equal). **Jianhua Xu:** conceptualization (equal), data curation (equal), investigation (equal), resources (equal), supervision (equal), writing – review and editing (equal).

## Funding

This study was supported by the Maoming Science and Technology Bureau of China (the project numbers are 2024kjcxLX007 and 2024kjcxLX008).

## Ethics Statement

The animal study protocol was approved by the Guangdong Medical Laboratory Animal Center (approval number: B202408‐3). The study adhered to the guidelines set by the committee.

## Conflicts of Interest

The authors declare no conflicts of interest.

## Supporting information


**Appendix S1:** fsn371472‐sup‐0001‐AppendixS1.docx.

## Data Availability

Data will be made available on request.
