# Supplementary material for: Exocarpium Citri Grandis‐Derived Extracellular Vesicle‐Like Particles for Accelerating Wound Healing via Regulating the Protein Expression on the VEGF/AKT Signaling Pathway
Source: Food Sci Nutr. 2026 Jan 28;14(2):e71472. doi: 10.1002/fsn3.71472 (PMC12848773; doi:10.1002/fsn3.71472)
Supplement: Supplementary file 1 — Appendix S1: fsn371472‐sup‐0001‐AppendixS1.docx. [file FSN3-14-e71472-s001.docx]

# Supplementary Materials

## 1. Supplementary Methods

### 1.1 Isolation and Purification of ECG-EVLP

Fresh Exocarpium Citri Grandis (ECG) fruits were processed following the protocol described in the main text, with the following additional details to ensure reproducibility. Centrifugation was performed using an Allegra X-30R refrigerated centrifuge equipped with a F0685 rotor, under the specified relative centrifugal forces at 4°C. Polyethylene glycol (PEG, MW 6000Da, Sigma-Aldrich) was dissolved in sterile phosphate-buffered saline (PBS, pH 7.4) and filtered through a 0.22 μm PES membrane (Millipore). After precipitation, the pellets were resuspended in PBS and filtered again through a 0.22 μm filter. Protein concentration was determined using the BCA Protein Assay Kit (Servicebio, G2026-200T). Each batch was prepared in triplicate (n = 3 technical replicates) using independent isolations.

### 1.2 Transmission Electron Microscopy (TEM)

ECG-EVLP samples were placed on carbon-coated copper grids (200 mesh) and negatively stained with 2% (w/v) uranyl acetate for 1 minute. Grids were air-dried at room temperature and imaged using a Hitachi HT7700 transmission electron microscope at 80 kV. At least five randomly selected fields were analyzed per sample.

### 1.3 Nanoparticle Tracking Analysis (NTA)

NTA was performed using a ZetaView PMX120 (Particle Metrix, Germany) at 25°C. The particle suspension was diluted to a final concentration of 4.4 × 10⁷ – 4.8 × 10⁷ Particles/mL. Each sample was measured in triplicate, with three 60-second videos captured for each replicate. The data were analyzed using ZetaView software (version 8.05.14).

### 1.4 Untargeted Metabolomics and Lipidomics

2-Amino-3-(2-chloro-phenyl)-propionic acid (Aladdin, 103616-89-3) was used as internal standards for Untargeted Metabolomics. Accurately add 300 µL acetonitrile: 2-Amino-3-(2-chloro-phenyl)-propionic acid (4 ppm) solution prepared with 0.1% formic acid (1:9, V / V). Raw data were converted to the mzXML format using MSConvert in the ProteoWizard package (v3.0.8789). Peak detection, filtering, and alignment were performed using the XCMS R package with the following parameters: bw = 2, ppm = 15, peakwidth = c(5, 30), mzwid = 0.015, mzdiff = 0.01, and method = “centWave.” Data normalization was performed using the total peak area to correct for systematic errors.

### 1.5 PKH26 Labeling and Cellular Uptake

ECG-EVLP (200 μg/mL) were labeled with 5 μM PKH26 (Merck, Germany) at 37°C for 60 min, followed by ultracentrifugation at 120,000 ×g (SW70Ti rotor) to remove free dye. Labeled vesicles were resuspended in PBS. HaCat and L929 cells were incubated with PKH26-labeled ECG-EVLP for 12 h. The nuclei were stained with Hoechst (10 μg/mL) and the cytoskeleton with FITC-phalloidin (2 μg/mL). Images were captured using a Leica Stellaris 5 confocal microscope under identical settings for all samples.

## 2. Reproducibility Statement

All experiments were conducted in at least three independent replicates using freshly prepared ECG-EVLP samples. For in vivo assays, three animals per group were included. Each figure in the main text represents the mean of independent experiments with standard deviation error bars. Experimental procedures and statistical analyses were performed using identical conditions to ensure reproducibility.
